# Highly efficient Co centers functionalized by nitrogen-doped carbon for the chemical fixation of CO_2_†

**DOI:** 10.1039/d0ra05238h

**Published:** 2020-11-23

**Authors:** Yuying Yang, Hong Li, Supeng Pei, Feng Liu, Wei Feng, Yongming Zhang

**Affiliations:** School of Chemistry and Chemical Engineering, Frontiers Science Center for Transformative Molecules, Shanghai Key Lab of Electrical Insulation and Thermal Aging, Shanghai Jiao Tong University No. 800 Dongchuan Rd., Minhang District Shanghai 200240 China ymzhang@sjtu.edu.cn; School of Chemical and Environmental Engineering, Shanghai Institute of Technology Shanghai 201418 China peisupeng@126.com; State Key Laboratory of Fluorinated Functional Membrane Materials, Dongyue Group Zibo 256401 China

## Abstract

CO_2_, the main greenhouse gas, has received considerable attention due to environmental issues. From a scientific perspective, CO_2_ as a cheap and abundant carbon source, could be applied in synthesizing more valuable chemicals such as urea, formic acid, and cyclic carbonates. However, the high bond energy of C=O (750 kJ mol^−1^) and the non-polarity property make CO_2_ molecules difficult to activate. In this paper, we have carefully designed a low-cost, stable and, most importantly, highly efficient Co-based heterocatalyst Co@N_*x*_C functionalized by nitrogen-doped carbon to activate CO_2_ molecules and convert it into cyclic carbonates. The CO_2_ conversion process could be triggered at very mild conditions (60 °C and 1 bar CO_2_). We carefully adjusted the nitrogen content in the carbon support to enhance the catalytic performance of Co centers *via* the interface effect. Consequently, the optimal catalyst displayed extraordinary activity toward the cycloaddition of CO_2_ with styrene oxide as high conversion (92%) and selectivity (>99%) were achieved in 4 h without byproducts.

## Abstract 2

A highly efficient Co@N_*x*_C functionalized heterocatalyst has been prepared to activate CO_2_ molecules and convert it into cyclic carbonates.

## Graphical abstract

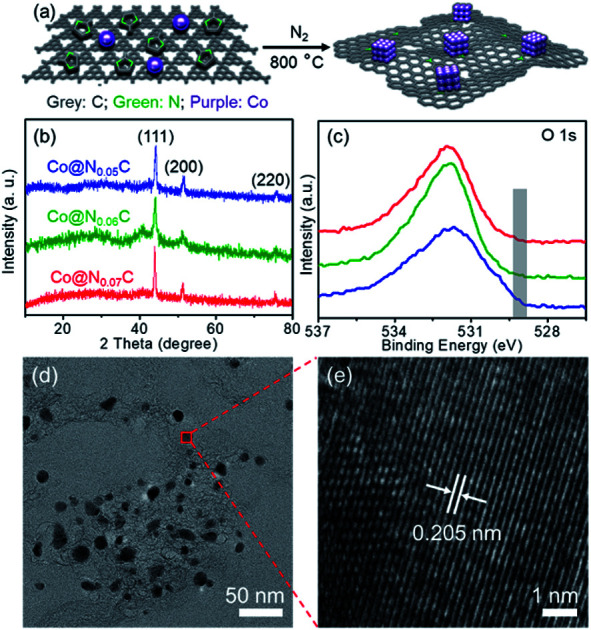

## 1. Introduction

The rigorous growth of the carbon dioxide volume in the atmosphere by fossil fuel consumption is a serious threat to the ecological system that causes greenhouse effect, leading to climate change, melting of glaciers, and rise of sea levels.^1–5^ Effective CO_2_ sequestration has thus been an important research topic that elevates greenhouse gas pressure, as well as provides useful products from a cheap carbon source.^6–8^ A technical difficulty of such process comes from the inert chemical nature of CO_2_, in which the reduction of CO_2_ to useful chemicals demands harsh conditions to activate CO_2_ molecules to undergo subsequent chemistry.^9,10^ High energy penalty is required to break the C=O bond. Processes such as electroreduction, photo reduction, and high temperature/high pressure hydrogenation are developed,^11–14^ which usually rely on noble metal catalysts that are expensive and complicated in the chemistry reaction loop. The low conversion of CO_2_ and poor selectivity of reduction products is still of high technical difficulty and needs to be addressed.^15^

In alternation, an easy cure of CO_2_ greenhouse gas problem is chemical fixation, which has high selectivity and good conversion yields.^16^ Cycloaddition fixation of CO_2_ with epoxides is of particular interest due to the wide range of applications of products of cyclic carbonates such as solvents, electrolytes, fine chemicals, and industrial lubricants.^13,17–21^ A variety of homo- and hetero-catalysts have been reported for CO_2_ cycloaddition. Homo-catalysts such as ionic liquids, metal complexes, and metal porphyrins, have a high TOF, but suffer from difficult separation.^22–24^ Hetero-catalysts such as porous polymers and metal–organic frameworks (MOFs) are advantageous for gas capture, catalysis activity, and easy separation; however, they suffer from harsh synthesis. Thus, the development of proper catalysts balancing synthetic costs and product separation gains is a key issue in effectively driving CO_2_ cycloaddition fixation.

A plausible pathway that addresses the above-mentioned catalysts paradox lies in the design of new heterogeneous catalytic systems. The assurance of high gas permittivity and catalysis activity requires a high specific inner surface area on which the catalysts should reside. The cost-effective concerns and separation simplicity call for a conventional material framework that is readily accessible, as well; as construction of new catalytic system in one-pot synthesis. These guidelines narrow the focus of mesoporous carbon materials, on which an inexpensive metal catalyst particles can be decorated in a hierarchical manner, thus providing an effective surface area and catalytic activity, as well as system stability and productivity. We show in this work that highly reactive ultrafine Co catalyst nanoparticles can be embedded in nitrogen-doped mesoporous carbon to homogeneously function as an active metal center and electron-withdrawing ligand quite. The activity of metal centers strongly depends on the coordination environment. In such a hetero-catalyst system, the surface electron density could be modified by nitrogen-doped carbon materials. An optimal catalyst Co@N_0.07_C is developed that leads to a 92% conversion yield within 4 hof the reaction time at 60 °C and CO_2_ gas pressure of 1 bar. No byproducts were detected, indicating high reactivity and selectivity.

## 2. Experimental section

### 2.1 Materials

CoCl_2_·6H_2_O (Sinopharm Chemical Reagent Co., Ltd), 2-methylglyoxaline (Sigma-Aldrich, Vetec™ reagent grade, 98%), urea (Sinopharm Chemical Reagent Co., Ltd), and styrene oxide (Aladdin, AR, 99.00%) were purchaed from companies indicated in brackets. Other substrates were purchased from Aladdin Industrial Corporation. All chemicals were directly used as-received without any further purification.

### 2.2 Synthesis of Co@N_*x*_C samples

CoCl_2_·6H_2_O (2 mmol; 475.86 mg) was dissolved in 100 mL of ethanol. After stirring, 8.947 g of carbon nitriding powder was added, and 16 mmol of 2-methyl imidazole (1.314 g) was added. The mixture was heated to 70 °C and vigorously stirred. The dried powder was placed into a crucible, capped, and placed in a muffle furnace. Nitrogen was continuously injected into the pot. The temperature was then increased to 400 °C at 2 °C min^−1^ for 2 h, and then heated to 800 °C for 4 h at 5 °C min^−1^. Eventually, it was naturally cooled to room temperature and removed to obtain the black product, which was then ground into a powder. Different proportions of CoCl_2_·6H_2_O and carbon nitride power were roasted with the program. While preparing Co@N_0.06_C and Co@N_0.05_C, only the dosage of carbon nitride powder was changed, *i.e.*, 17.849 and 44.735 g, respectively. There was no subsequent purification process for all samples.

## 3. Results and discussion

### 3.1 The synthesis and characterization of as-prepared Co@N_*x*_C samples

**Fig. 1:**
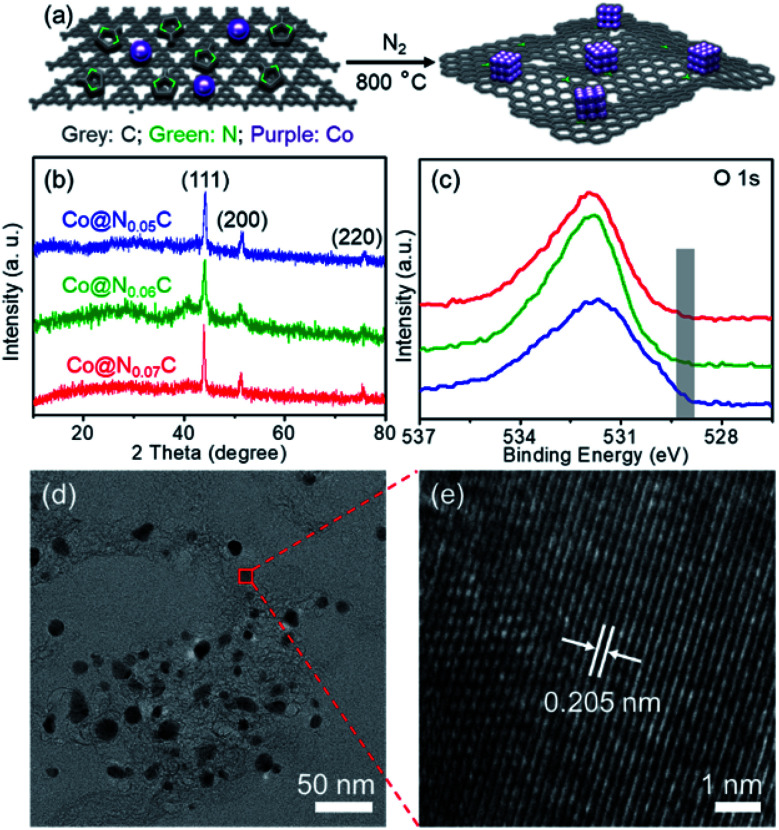
(a) Illustration of the synthesis process of the Co@N_*x*_C sample derived from Co salt, 2-methyl imidazole and carbon nitride; (b) powder XRD patterns (JCPDS No: 15-0806) and (c) the O 1s XPS of the Co@N_*x*_C samples; (d) a typical TEM image of the Co@N_0.07_C sample and (e) an HRTEM image of a randomly selected Co nanoparticle from (d).

As shown in Fig. 1a, a nanoconfined method was applied to develop new catalysts from cheap Co salt precursors. 2-Methyl imidazole and carbon nitride were used as the soft template *via* the high temperature carbonization process under a N_2_ atmosphere. High-temperature treatment leads to Co nanocrystallization, which is confined in precursor support. The nitride moieties could interact with Co nanoparticles to inhibit the formation of oversized crystals and serve as kink spots to pin Co nanocrystals on carbon support materials. The three catalysts are named after the ratio of nitrogen mole fraction (Table S1†). Powder X-ray diffraction (XRD) of Co@N_*x*_C samples was collected to confirm the formation of Co nanoparticles, and the specific peaks of Co (111), (200) and (220) peaks were observed (Fig. 1b). There were no diffraction peaks from cobalt nitrides, carbides, or oxides. Different precursor composition leads to slightly different crystallization details. As seen from XRD peaks, Co@N_0.07_C displays the best crystal coherence length in length of 0.239 nm, as estimated from the sharp (111) peak using Scherrer's equation. The surface electronic structure of the Co nanocrystal catalyst was studied using X-ray photoemission spectroscopy (XPS, Fig. 1c, S1–S3† and Table S1†). The as-prepared catalyst samples appeared as fluffy carbon foams, which showed a high BET surface area of up to 700.6 m^2^ g^−1^ for the Co@N_0.07_C sample. A mesoporous structure was revealed by scanning electron microscopy (SEM, Fig. S4†). N_2_ adsorption–desorption isotherms (Fig. S5†) result the porous structure of the catalysts. After calculation, the high specific surface areas of the catalysts were listed in Table S2.† The morphology of Co nanoparticles in a mesoporous carbon support was characterized by a transmission electron microscope (TEM, Fig. 1d). Co nanoparticles with an average diameter of 11.5 nm were well dispersed in a carbon matrix (Fig. S6†). The high-resolution TEM image of Co@N_0.07_C (Fig. 1e) of a single Co nanoparticle showed a lattice spacing of 0.205 nm, coming from to the (111) plane of metallic cobalt lattice. The contents of Co metals are all approximately 22 wt% as tested by ICP analysis (Table S3†); the value varied when the precursor loading changed.

### 3.2 The synthesis and characterization of as-prepared Co@N_*x*_C samples

**Fig. 2:**
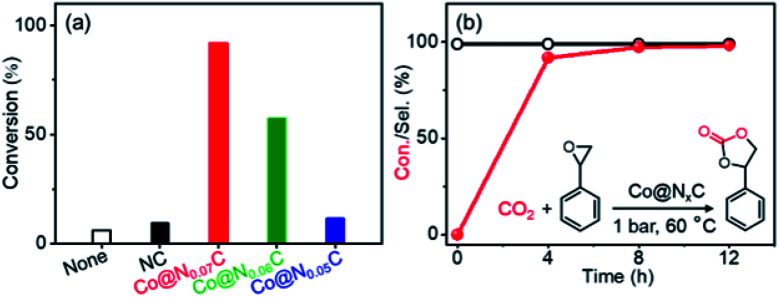
(a) Conversions of styrene oxide with CO_2_ over different samples; (b) time course of the conversion of styrene oxide and the selectivity of the target product phenylethylene carbonate in the presence of the Co@N_0.07_C catalyst. Reaction conditions: 1 mmol styrene oxide, 50 mg catalyst, 0.2 equivalent TBAB, 2 mL acetonitrile, 1 atm CO_2_ (balloon), 60 °C, 4 h.

The cycloaddition of styrene oxide with CO_2_ was chosen as a model reaction to evaluate the catalytic performance of Co@N_*x*_C catalysts. As shown in Fig. 2a, only a small amount of the substrate material (about 6.2%) was converted into the target product phenylethylene carbonate when the control catalyst tetrabutyl ammonium bromide (TBAB) was used under a mild condition (1 bar CO_2_ (balloon), 60 °C). Furthermore, slightly improved conversion (about 9.6%) was achieved over the metal-free sample NC. The new catalyst developed, Co@N_0.07_C, exhibited an extraordinarily high catalytic activity, and the conversion yield of 92% with excellent selectivity (>99%) under the same condition. However, when the content of nitrogen in the support decreased, the conversion yield dropped to 57.2% using Co@N_0.06_C, and then further decrease to 11.4% using Co@N_0.05_C. The reaction kinetics was probed using the Co@N_0.07_C catalyst, and a complete conversion was achieved in 12 h, as shown in Fig. 2b. The decrease of reaction rate is due to the decreased concentration of styrene oxide.

To further study the general catalytic applications of the Co@N_0.07_C, a variety of ethylene oxide substituted with both electron-donating (–CH_3_ and –C_2_H_5_) and electron-withdrawing (–Cl, –OPh) functional groups were used to in CO_2_ cycloaddition reaction under the standard conditions. As shown in Table 1, the Co@N_0.07_C sample generally afforded high selectivity (>99%) of the substituted ethylene oxide and good conversion (>71%) toward the corresponding cyclic carbonates (entries 1–4 in Table 1). The relatively low conversion of 4 was attributed to the steric effect and the fat that the –OPh group blocked the pre-adsorption of the epoxy group on the surface Co centers to obstruct the reaction (entry 4 in Table 1). Generally, the Co@N_0.07_C catalyst possessed excellent catalytic ability toward the cycloaddition of CO_2_ with epoxides. In addition to the excellent tolerance to different functional groups, the Co@N_0.07_C catalyst displayed good stability. After five continuous reaction test cycles, the catalytic activity of the Co@N_0.07_C sample was kept almost unchanged in conversion yield and selectivity (Fig. S7 and Table S4†), and the chemical structure nearly remained the same as the XRD pattern (Fig. S8†).

**Table 1:** The CO_2_ cycloaddition with a series of epoxide derivatives in the presence of the Co@N_0.07_C catalyst^a^

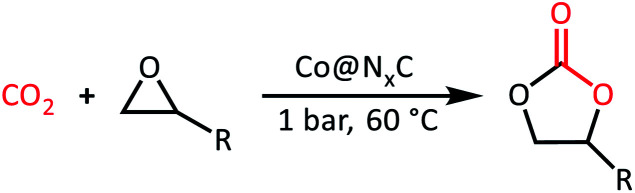				
Entry	Substrate	Product	Con. (%)	Sel. (%)
1	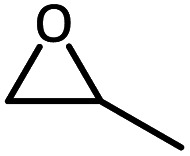	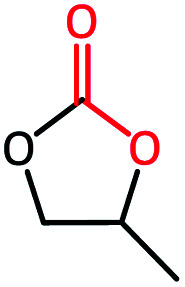	>99	>99
2	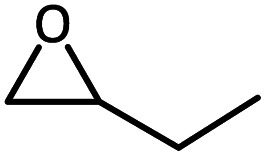	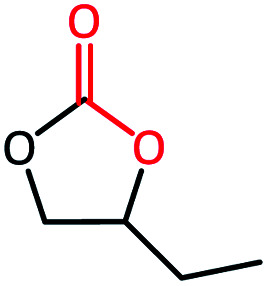	>99	>99
3	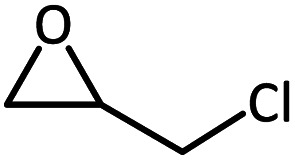	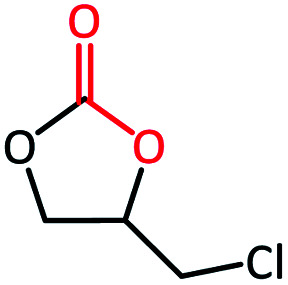	84	>99
4	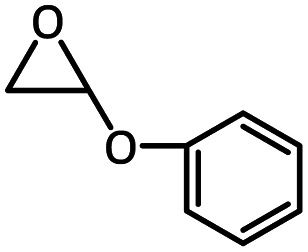	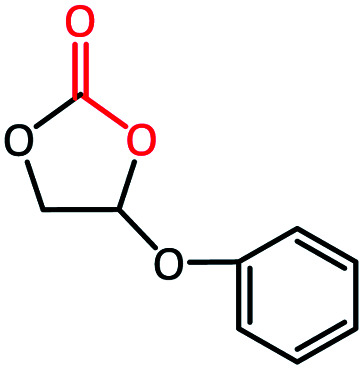	71	>99

a Reaction conditions: 1 mmol substrate, 50 mg Co@N_*x*_C catalyst, 0.2 equivalent TBAB, 2 mL acetonitrile, 1 atm CO_2_, 60 °C, 12 h.

### 3.3 Electronic structure analysis and exploration of mechanics

**Fig. 3:**
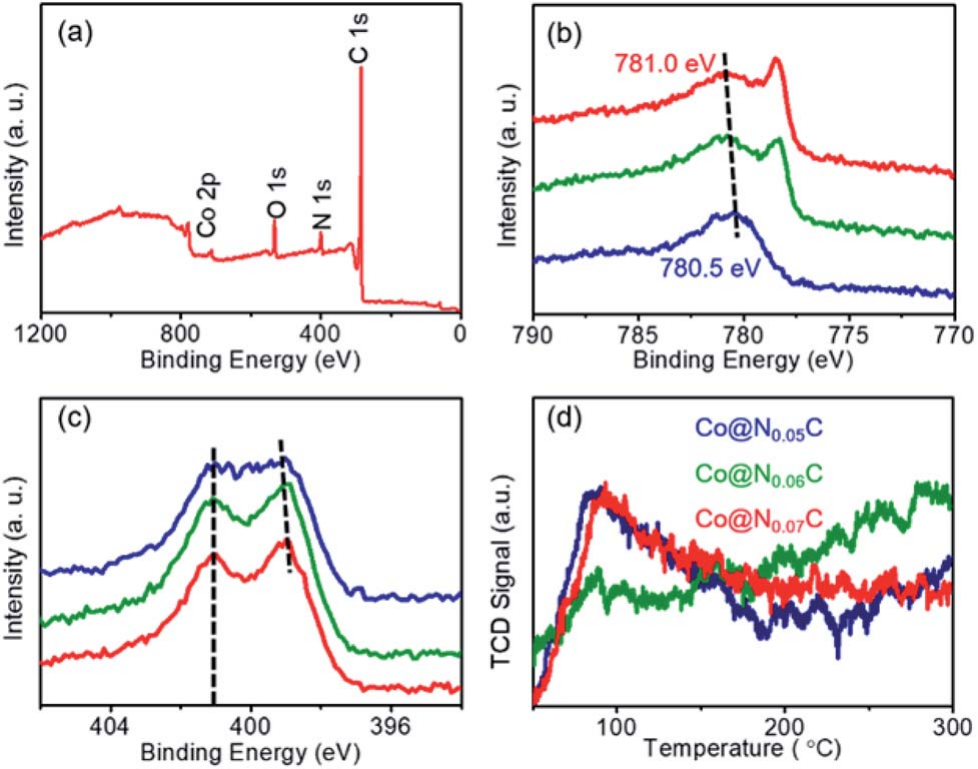
(a) The XPS spectra of Co@N_0.07_C; (b) Co 2p; (c) N 1s XPS spectra; (d) the CO_2_-TPD spectra of Co@NxC samples.

We further look into the interaction between Co nanoparticles and nitrogen-doped carbon using XPS spectra (Fig. 3a). As shown in Fig. 3b, when more nitrogen-doped carbon support is introduced, the Co 2p XPS peaks gradually shifts to the high-energy zone, indicating that electron transfers to the nitrogen-doped carbon. The N 1s XPS spectra not only showed the peaks with the binding energy located at about 398.2 and 401.1 eV, respectively, corresponding to pyridinic nitrogen and graphitic nitrogen (Fig. S1–S3†), but also suggested a shift of the pyridinic N 1s XPS peaks, as shown in Fig. 3c. Such a shift in the binding energy of N directly demonstrates the role of nitrogen-doped carbon support as the electron acceptor. As a result, the electron transfer between the Co nanoparticles and the carbon support could give rise to a relatively high electron density state of the carbon support surface and benefitted the adsorption of CO_2_ molecules, which could be easily examined by the experimental results of CO_2_ temperature programmed desorption (CO_2_-TPD) (Fig. 3d).

**Scheme 1:**
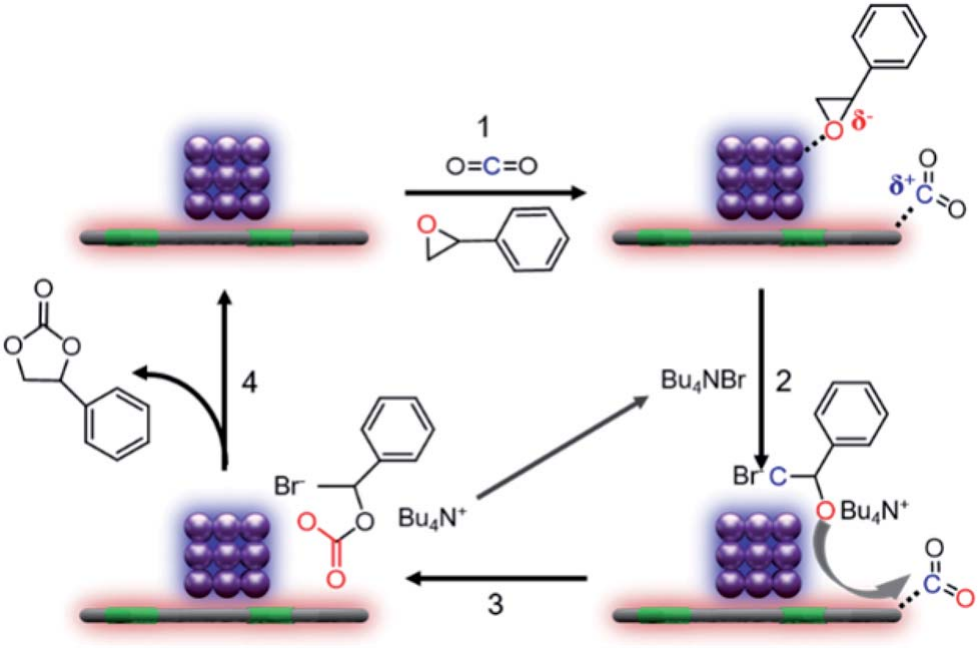
Proposed mechanism for the CO_2_ cycloaddition with styrene oxide over the Co@N_*x*_C catalyst with the assistance of TBAB.

The mechanism for CO_2_ cycloaddition with epoxides using tetrabutyl ammonium bromide (TBAB) was proposed by Caló who pointed out that “the bulkiness of the tetrahedral ammonium ion forces the bromide or iodide ion away from the cation, and this less electrostatic interaction would render these anions more nucleophilic”, suggesting the key importance of TBAB in the reaction process.^25^ However, in our catalytic system, the conversion over Co@N_0.07_C and TABA is 14.8 times of that with the presence of only TABA, thus highlighting the crucial role of our nanocatalyst. In addition, electron-rich and electron-deficient areas at the boundary of metal–semiconductor nano-composites could function as Lewis acid and base pairs,^26,27^ which facilitate the fixation of CO_2_ and activation of epoxides.^28–30^ So, considering the actual situation, we proposed the mechanism of CO_2_ cycloaddition with styrene oxide at the enhanced density and strength of the Lewis acid–base sites in our catalytic system as described in Scheme 1. The first step is known to be the substrate molecules absorbed on the surface of the heterocatalyst, that is, styrene oxide absorbed on the surface of the electron-deficient Co nanoparticles and CO_2_ molecules on the electron-rich nitrogen-doped carbon support. Then, in step two, after a nucleophilic attack by the bromide ion, the ring of the epoxide opened. Finally, activated 
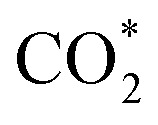
 molecules would bind the oxy anion species to afford the corresponding cyclic carbonate (step 3).

## 4. Conclusions

To conclude, a stable, cheap and highly efficient heterocatalyst, Co@N_*x*_C, was developed *via* a simple nano composite approach. The electron-deficient state of Co centers functionalized by the nitrogen-doped carbon displayed excellent catalytic performance toward the CO_2_ cycloaddition with epoxides as 92% conversion and almost 100% selectivity of the target product achieved at 4 h (60 °C and 1 atm CO_2_ gas), and the general applicability and recycling test showed the potential application value. We believed that this work not only provided a new idea to develop the highly efficient heterocatalysts but also advanced the scientific research toward CO_2_ related issues.

## Conflicts of interest

The authors declare that they have no competing interests.

## Supplementary Material

RA-010-D0RA05238H-s001
