# Supplementary material for: Highly efficient Co centers functionalized by nitrogen-doped carbon for the chemical fixation of CO2
Source: RSC Adv. 2020 Nov 23;10(69):42408–12. doi: 10.1039/d0ra05238h (PMC9057963; doi:10.1039/d0ra05238h)
Supplement: RA-010-D0RA05238H-s001 [file RA-010-D0RA05238H-s001.pdf]

Electronic Supplementary Material (ESI) for RSC Advances.
This journal is © The Royal Society of Chemistry 2020

Supporting Information

Highly efficient Co centers functionalized by nitrogen-doped carbon for the chemical fixation of CO_2

Yuying Yang [a], Hong Li [a], Supeng Pei [b], Feng Liu [a], Wei Feng [c], and Yongming Zhang [a,*]

[a] School of Chemistry and Chemical Engineering, Frontiers Science Center for Transformative Molecules, Shanghai Key Lab of Electrical Insulation and Thermal Aging, Shanghai Jiao Tong University, No. 800 Dongchuan Rd., Minhang District, Shanghai 200240, China.

[b] School of Chemical and Environmental Engineering, Shanghai Institute of Technology, Shanghai 201418, China.

[c] State Key Laboratory of Fluorinated Functional Membrane Materials, Dongyue Group, Zibo 256401, China.

Supplementary Methods

Materials and Methods

Materials

$CoCl_2 \cdot 6H_2O$ (Sinopharm Chemical Reagent Co., Ltd), 2-Methylglyoxaline (sigma-aldrich, Vetec™ reagent grade, 98%), urea (Sinopharm Chemical Reagent Co., Ltd); styrene oxide (Aladdin, AR, 99.00%). Other substrates were purchased from Aladdin Industrial Corporation. All the chemicals were directly used as received without any further purification.

Synthesis of Co@N_xC samples

2 mmol $CoCl_2 \cdot 6H_2O$ (475.86 mg) was dissolved in 100 mL ethanol. After stirring, 8.947 g of carbon nitriding powder was added, and 16 mmol 2-methyl imidazole (1.314 g) was added. Heat the mixture to 70 °C and stir it vigorously. The dried powder was placed into a crucible, capped, and put into a muffle furnace. Nitrogen was continuously injected into the pot. The temperature was raised to 400 °C at 2 °C /min for 2 h, and then heated to 800 °C for 4 h at 5 °C /min. Eventually, it was cooled naturally to room temperature and removed to obtain the black product, which was then ground into a powder. Different proportions of Co@N_xC were roasted with the program. During the preparation of Co@$N_{0.06}$C and Co@$N_{0.05}$C, only the dosage of carbon nitride powder was changed, which were 17.849 g and 44.735 g, respectively. There was no subsequent purification process for all samples.

The CO_2 cycloaddition with styrene oxide and its derivatives

Prepare 0.1201 g styrene oxide (1 mmol) in a flask, add 50 mg Co@N_xC catalyst and 0.0645 g TBAB (0.2 mmol), and add 2 mL acetonitrile. After three times of washing with CO_2, the reactants were reacted for 12 hours at a temperature of 60 ℃ in an atmosphere of 1 ATM CO_2. Samples were collected for GC-MS analysis after the reaction time of 4 h and 8 h separately. The samples of ethylene oxide substituted

reaction 1, 2, 3 were analyzed by ^1H NMR.

Characterization

Scanning electron microscopy (SEM) observations were recorded on a Nova NanoSEM 230 field emission scanning electron microscope (FEI, USA). The transmission electron microscopy (TEM) and high-resolution transmission electron microscopy (HRTEM) images were taken by a JEM-2100F microscope operated at an acceleration voltage of 200 kV. Powder X-ray diffraction (XRD) patterns were performed on a Bruker D8 Advance X-ray diffractometer with Cu Kα radiation (λ = 1.5418 Å). X-ray photoelectron spectroscopy (XPS) experiments were performed at a Kratos Axis Ultra DLD spectrometer and ESCALAB 250 photoelectron spectrometer (Thermo Fisher Scientific). The surface area was determined by a multipoint Brunauer-Emmett-Teller (BET) method, using an ASAP 2460 surface area and porosimetry analyzer. The high-angle annular dark-field scanning transmission electron microscopy (HAADF-STEM) images were taken by a Titan 80-300 scanning/transmission electron microscope operated at 300 kV, equipped with a probe spherical aberration corrector. The sample The qualitative and quantitative analysis of each substance in the reaction system is by means of QP2010E gas chromatography - mass spectrometry (GC-MS) and AVANCE III HD 400 nuclear magnetic resonance (^1H NMR).

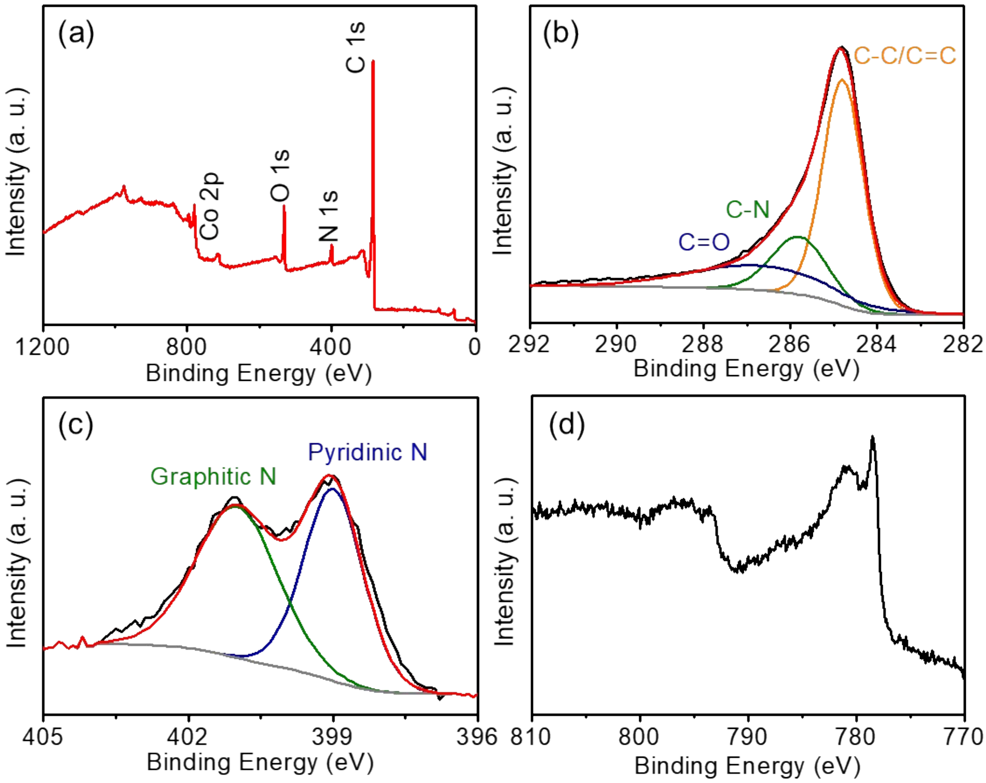

Figure S1. (a) The XPS survey spectrum, (b) C 1s, (c) N 1s and (d) Co 2p XPS spectra of Co@$N_{0.07}$C.

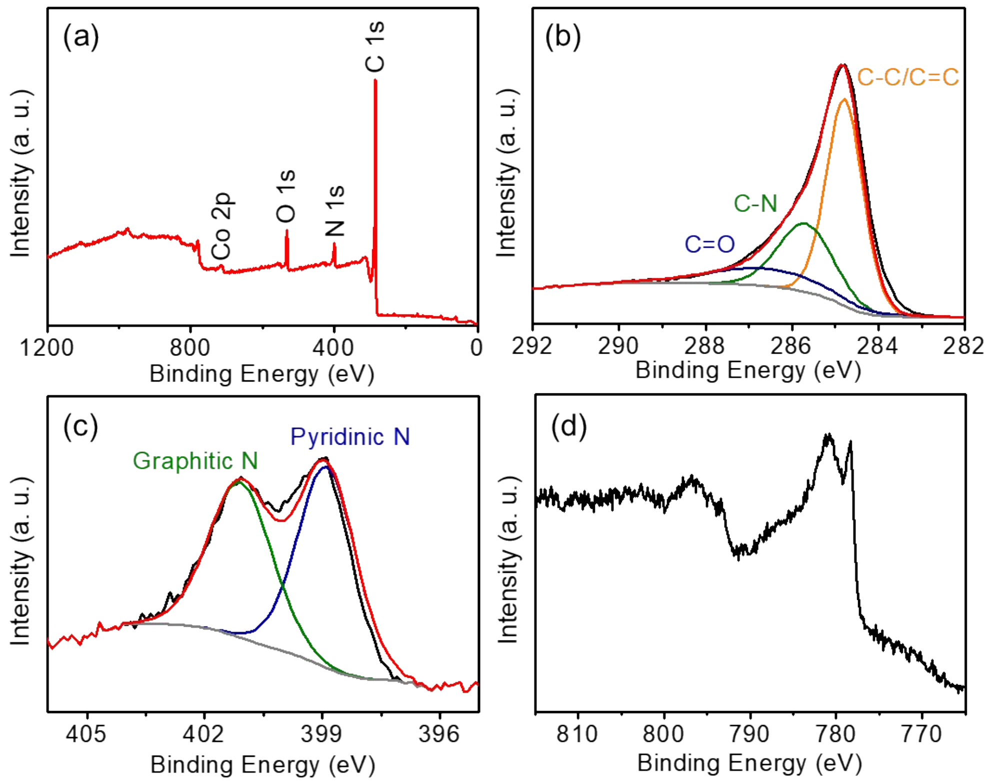

Figure S2. (a) The XPS survey spectrum, (b) C 1s, (c) N 1s and (d) Co 2p XPS spectra of Co@$N_{0.06}$C.

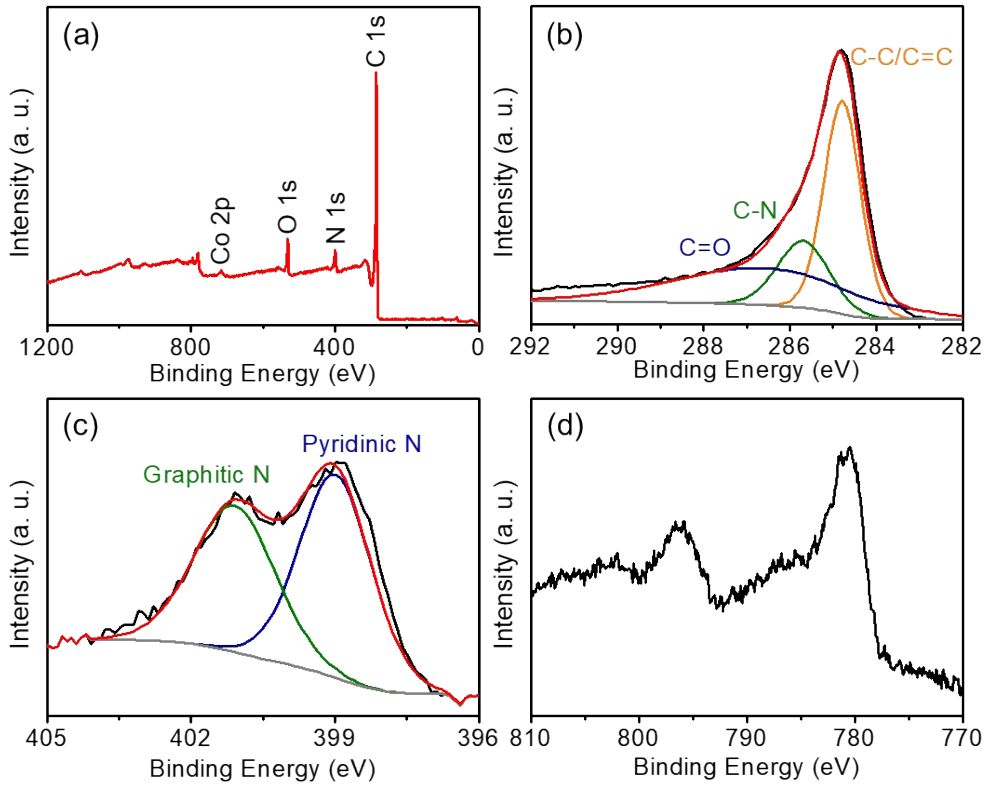

Figure S3. (a) The XPS survey spectrum, (b) C 1s, (c) N 1s and (d) Co 2p XPS spectra of Co@$N_{0.05}$C.

Table S1. Elemental analysis of Co, C, N and O based on the XPS analysis results.

Sample	Co	C	N	O
	Atomic percentage (at. %)			
Co@$N_{0.07}$C	6.09	81.58	6.49	5.83
Co@$N_{0.06}$C	5.09	82.79	6.23	5.89
Co@$N_{0.05}$C	6.67	79.64	4.28	9.41

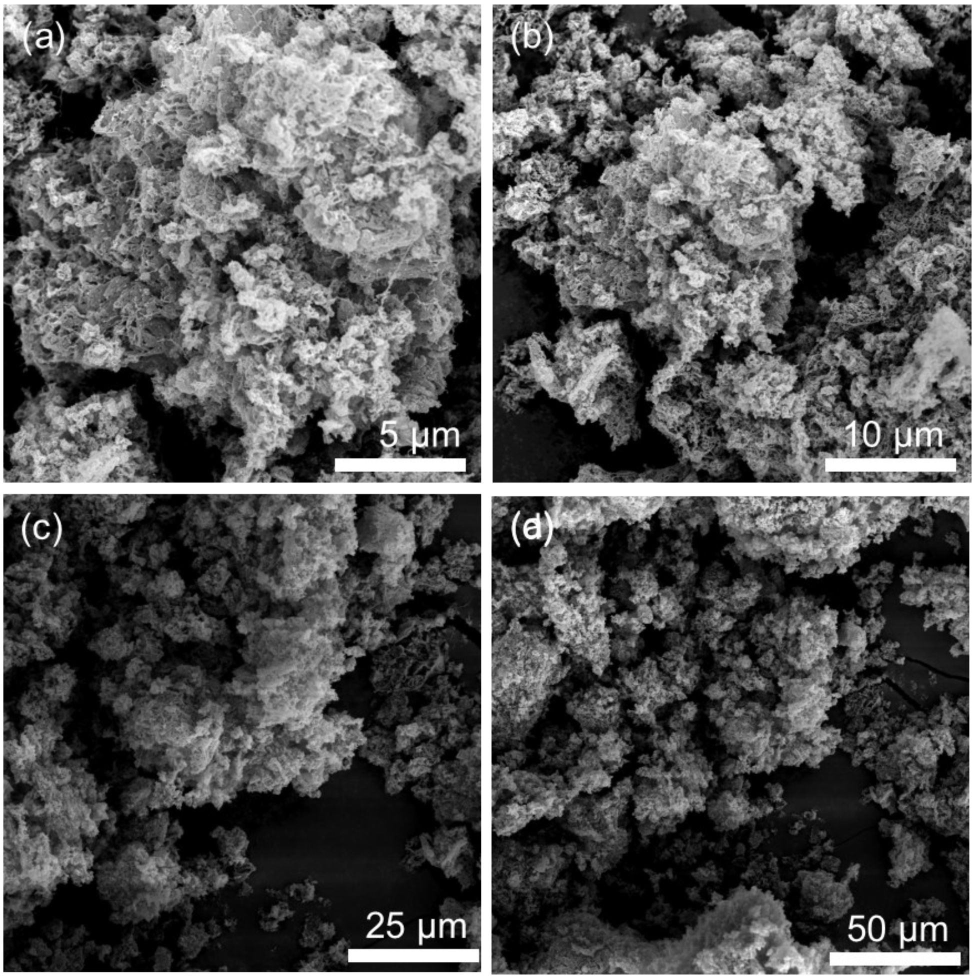

Figure S4. SEM images of the as-prepared Co@$N_{0.07}$C sample at different scales.

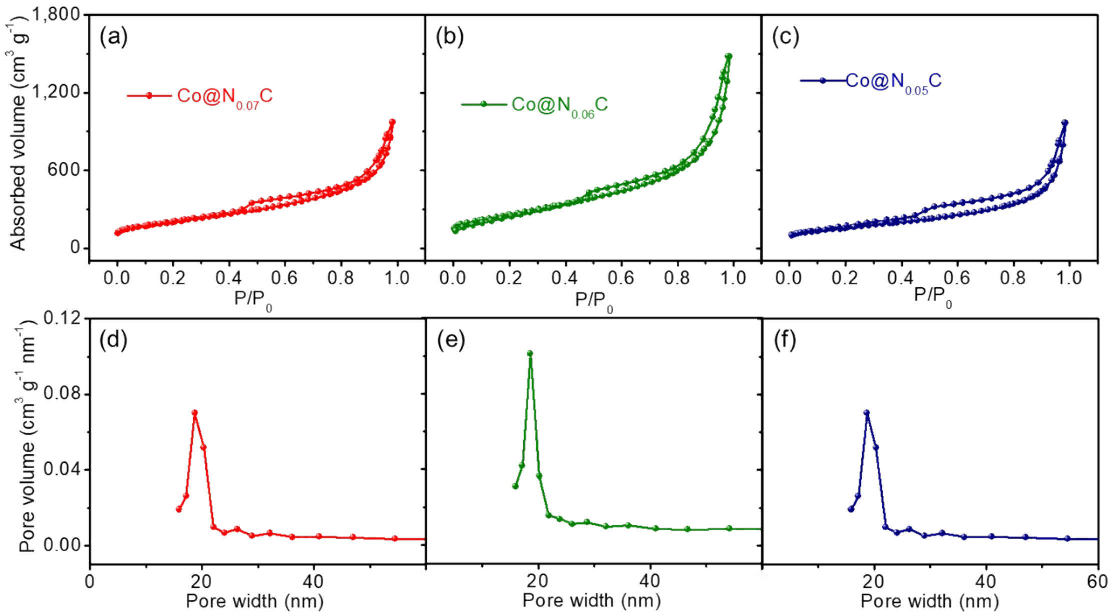

Figure S5. (a-c) N_2 sorption isotherm curves and corresponding (d-f) pore size distribution of the Co@NC samples.

Table S2. N_2 sorption data for the Co@N_xC samples.

Sample	Surface (m^2 g^{-1})	Pore volume (cm^3 g^{-1})
Co@$N_{0.07}$C	700.6	1.36
Co@$N_{0.06}$C	632.6	1.40
Co@$N_{0.05}$C	300.7	2.18

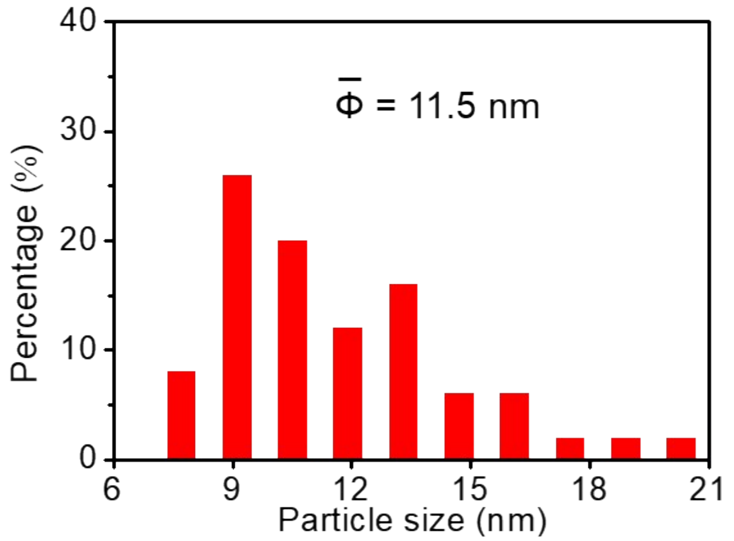

Figure S6. The particle size distribution of Co nanoparticles in the Co@$N_{0.07}$C sample.

Table S3. The Co content in the Co@NxC samples based on the ICP results

Sample	Co content (wt. %)
Co@$N_{0.07}$C	22.21
Co@$N_{0.06}$C	23.00
Co@$N_{0.05}$C	29.89

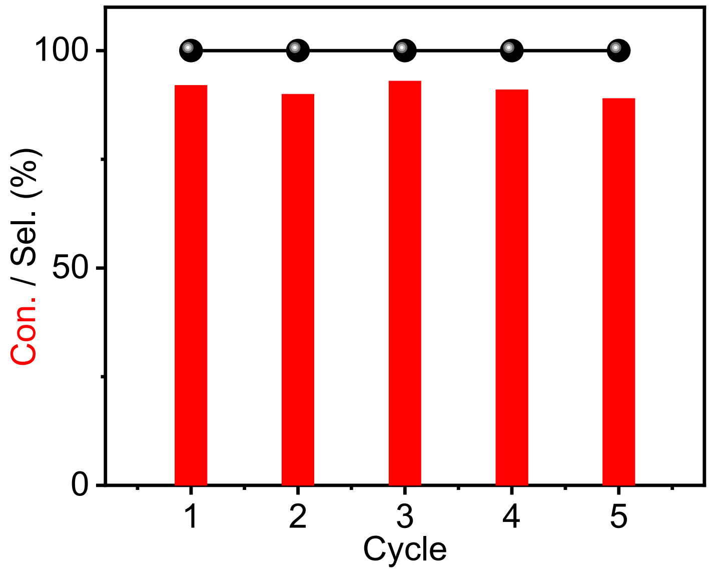

Figure S7. The recycle test of Co@$N_{0.07}$C catalyst in the reaction of CO_2 cycloaddition with styrene oxide.

Table S4. The recycle test of Co@$N_{0.07}$C catalyst in the reaction of CO_2 cycloaddition with styrene oxide.

Cycle	Con. (%)	Sel. (%)
1	92	>99
2	90	>99
3	93	>99
4	91	>99
5	89	>99

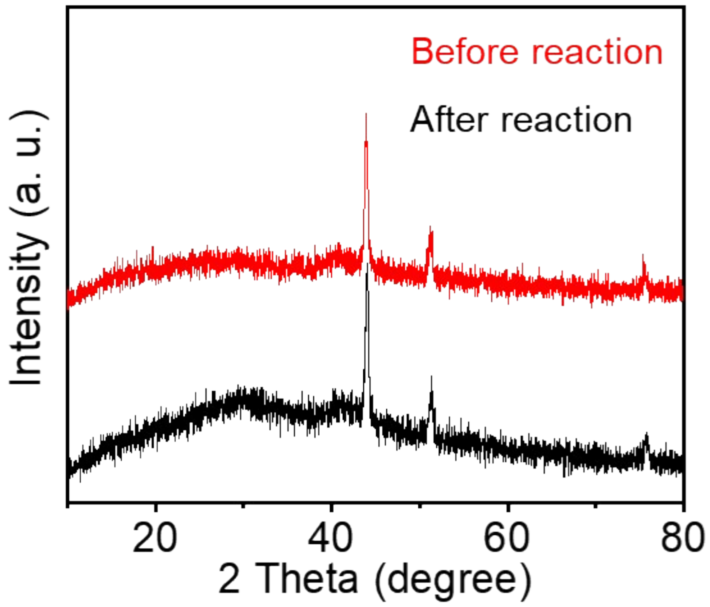

Figure S8. Powder XRD patterns of the $Co@N_{0.07}C$ catalyst before and after the reaction.

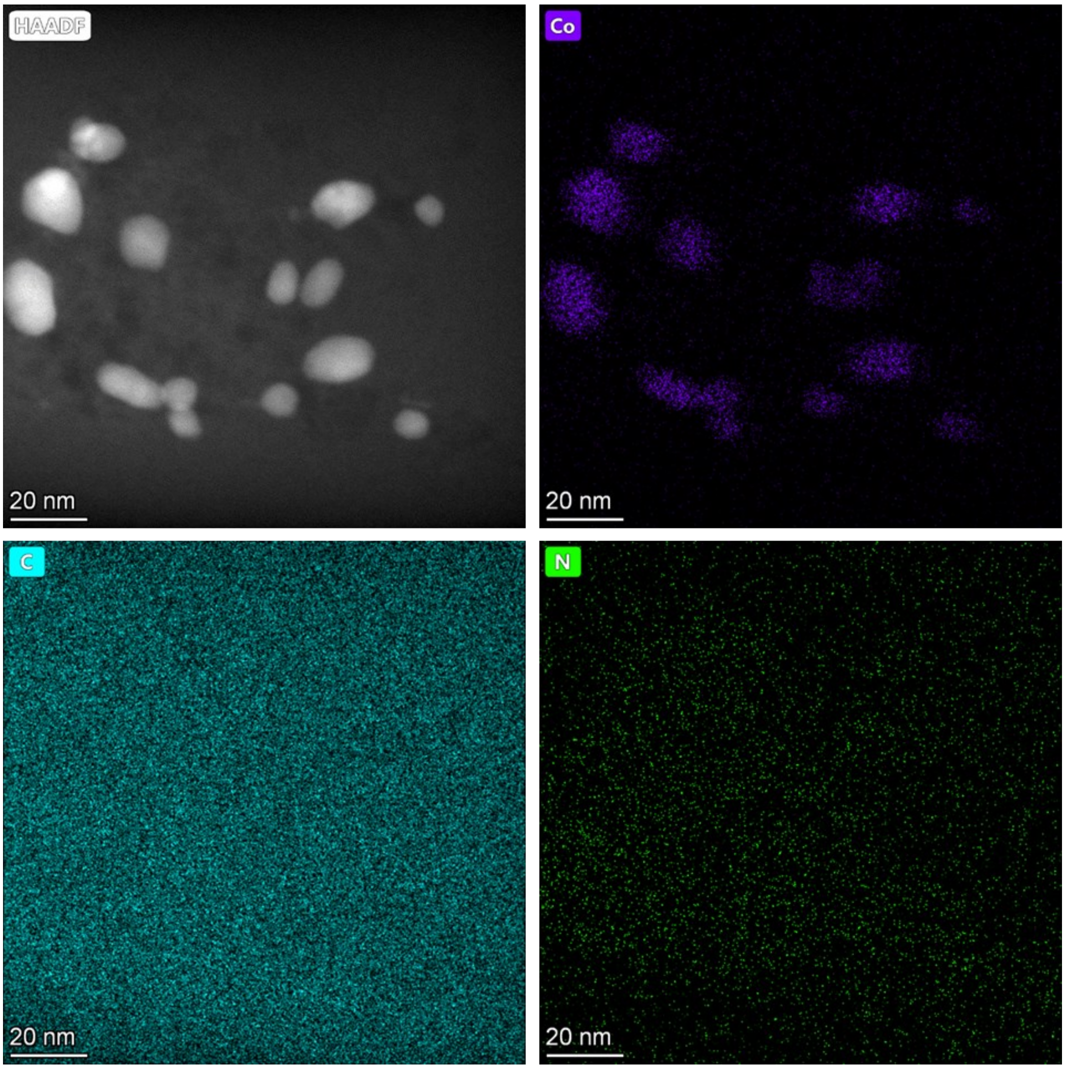

Figure S9. A HAADF image of $Co@N_{0.07}C$ and the corresponding element mapping of Co, C and N respectively.
